# Estimating discharge dates using routinely collected data: improving the preparedness of parents of preterm infants for discharge home

**DOI:** 10.1136/archdischild-2016-310944

**Published:** 2016-10-03

**Authors:** Peter J Fleming, Jennifer Ingram, Debbie Johnson, Peter S Blair

**Affiliations:** Centre for Child and Adolescent Health, University of Bristol, Bristol, UK

**Keywords:** Neonatology, Audit, Discharge Planning, Length of Stay

## Abstract

The length of stay for moderately preterm infants has progressively become shorter in the UK in recent years but staff still commonly inform parents that their baby will go home around their estimated date of delivery (EDD). Parents need as much notice as possible to prepare for the discharge of their baby, and to gain the necessary skills and knowledge to care for their infant safely. We report the use of routinely collected neonatal data to develop and implement a simple centile chart for date of discharge from hospital, which allows staff and parents to predict the likely discharge date more accurately for preterm infants, most of whom now go home more than 3 weeks before their EDD. This information allows better and timelier planning for discharge of such infants, by parents and staff.

**What is already known on this topic?**

- Most preterm infants are discharged to home well before their original estimated date of delivery (EDD).
- Neonatal staff still commonly inform parents that their infant is likely to go home around the time of the EDD.
- Preparation of parents for the discharge of their infant is commonly left until late in the course of the infant's stay in hospital.

**What this study adds?**

- The clinical database routinely used in UK neonatal units allows the preparation of simple individualised centile charts for length of stay for infants in each neonatal network at each gestation.
- This allows staff and parents to know well in advance approximately when each infant is likely to be discharged.

## Background

Having a preterm baby places great strain on families, and preparing for the day when the baby comes home is a time-consuming and emotionally fraught activity. Parents, hospital and community staff need as much warning as possible of the likely date that a preterm infant will go home to ensure that all aspects of preparation are completed in good time, and not rushed at the last minute when a decision is made to discharge the baby. Historically, most paediatricians and neonatal nurses have told parents of preterm infants that the baby would probably go home around the time the baby would have been due to be born, the estimated date of delivery (EDD). Despite considerable evidence that, for many moderately preterm infants the length of hospital stay has been progressively shortening over recent years.^1^ ^2^ In a survey of neonatal units in the UK in 2011, we found that most staff still used the EDD as the likely discharge date. Several recent studies have reported complex models using detailed information on infant condition and pathophysiology that allow estimation of likely length of stay (and thus date of discharge) for preterm infants.^1–4^ The main purpose of such models has been to allow generic comparison of standards and outcomes of care in different institutions and thus to raise overall standards rather than at the individual level. We report a simple approach to estimation of length of stay for preterm infants that is designed primarily to prepare staff and parents for the baby's discharge rather than to compare practices in different institutions. This approach uses data routinely collected by the ‘Badger.net’ database used by almost all neonatal units in the UK.

## Methods

Four local neonatal units (LNUs) in the Southwest region of England participated in a study that aimed to improve parental self-efficacy for parents of preterm infants.^5^ This study required that we estimate as accurately as possible, early in the hospital stay, the likely date of discharge for participating infants.

In this neonatal network, all infants born in an LNU at <27 weeks’ gestation are routinely transferred to a (level 3) neonatal intensive care unit (NICU) immediately after delivery, and thus very few such infants receive early care in a (level 2) LNU. As care in LNUs was the main focus of the study, we only included infants born in the study LNUs between 27 weeks 0 days and 33 weeks 6 days gestation. From the routine neonatal database (Badger.Net; Clevermed), we collected data on lengths of stay for all infants in the target gestational age range over a 2-year period immediately preceding our planned intervention in 2013 who were born in and discharged to home from the same LNU. We did not exclude infants who had an intervening period in a regional NICU as long as they survived and were discharged to home from the LNU of their birth. From the information routinely collected on the ‘Badger.net’ database, we obtained information on gestation at birth (in weeks and days), and age in days at the time of discharge to home for all infants in the target gestational age range. These data were exported into an Excel spreadsheet, aggregated into whole weeks of gestation, and charts were produced to identify the 50th, 75th and 90th centiles of lengths of stay for infants at each gestation.

## Results

**Figure 1:**
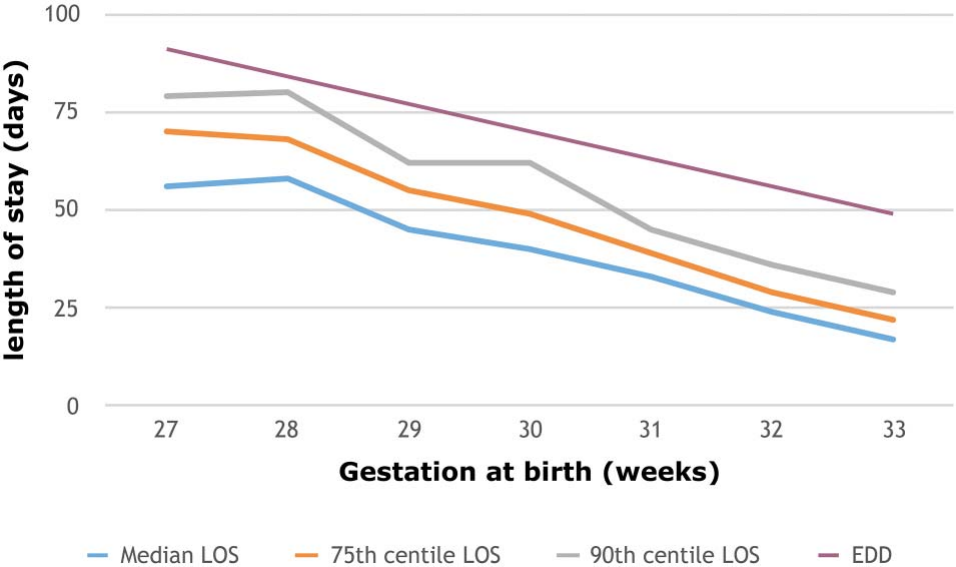
Median, 75th centile and 90th centile for lengths of stay (LOS) and estimated date of delivery (EDD) for preterm infants, 2011–2013.

Overall 531 preterm infants met the criteria and were included in the study, with 26 infants born at 27 weeks’ gestation, rising to 171 born at 33 weeks’ gestation. The 50th (median), 75th and 90th centiles for lengths of stay, together with the duration of stay representing the EDD for each gestation, are shown in figure 1.

The EDD is consistently 3–4 weeks later than the median length of stay for each gestational age and for most ages 1–2 weeks later than the 90th percentile when the large majority of infants have gone home.

At each gestation, a small number of infants, who suffered a more complex pathway than the majority, had a much longer hospital stay than most. This led to greater variation in the 90th centile than other centiles for length of stay, though even for the 90th centile the discharge date was earlier than the EDD at all gestations. The 75th centile for age at discharge was more consistent, being between 36 and 37 weeks postmenstrual age (gestation plus postnatal age), for almost all gestations.

In this population of 531 preterm infants, 75% went home 3 weeks or more before the date on which the baby was originally anticipated to have been delivered (the EDD).

The range of lengths of stay between the 50th and 75th centiles was greater for gestations between 27 and 30 weeks (10–14 days) than that for infants of 31–33 weeks' gestation (5–6 days).

**Figure 2:**
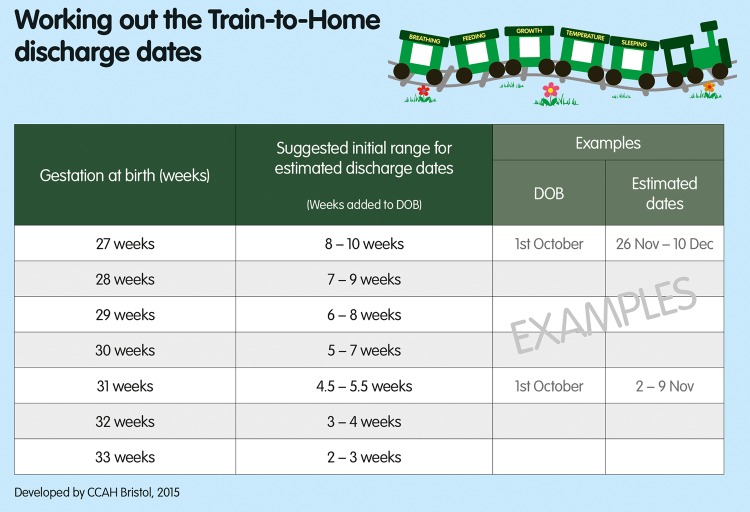
The simple chart used to help medical and nursing staff calculate the range of estimated discharge dates for babies of each gestation.

Figure 2 shows the simple chart that is used to help medical and nursing staff to calculate the smoothed 50th and 75th centiles for babies of each gestation.

We needed a consistent and usable chart that would enable medical and nursing staff to give parents within 7–10 days after an infant's birth an estimated range of dates for discharge from hospital to home. We used the 75th centile, which was smoothed to minimise the week-by-week variations. To make the chart usable and avoid staff making time-consuming calculations for each baby, we included estimated lengths of stay in whole weeks for infants of each gestation apart from 31 weeks for which we gave the range of 4.5–5.5 weeks. For simplicity and ease of use, we gave a 2-week range of dates for the less mature infants, and 7 days for the more mature infants, each representing approximately the range between the 50th and 75th centiles. Parents and staff were informed that by the end of this time period there was a 75% probability that their baby would have been discharged.

In semistructured qualitative interviews with 21 parents as part of the main study,^5^ parents welcomed this innovation, and reported that ‘the dates prepared them for going home’. Having more accurate dates helped them to make plans for the time of discharge while accepting the impossibility of knowing in advance about unexpected problems that might delay discharge.

## Discussion

The estimated dates of likely discharge in this study showing that most infants of 27–33 weeks' gestation will have been discharged home before 37 weeks post menstrual age are in line with other recent studies.^1^ ^4^ The earlier discharge of preterm infants leaves less time for parents to prepare and gain the necessary skills and understanding of the needs of such infants after discharge.

The use by almost all neonatal units in the UK of a standard database (Badger.net) from which recent lengths of stay for infants of each gestation can easily be produced from routinely collected data for each neonatal network means that in each neonatal unit recent, locally generated data on likely lengths of stay for preterm infants can be produced and regularly updated.

We suggest that the use of such locally generated charts may be of value in allowing staff and parents to predict approximately when preterm infants are likely to be discharged home, allow for individual tailoring of discharge dates and thus to ensure adequate and timely preparation for discharge.
